# The Shorter the Better: Reducing Fixed Primer Regions of Oligonucleotide Libraries for Aptamer Selection

**DOI:** 10.3390/molecules14041353

**Published:** 2009-03-27

**Authors:** Weihua Pan, Gary A. Clawson

**Affiliations:** 1 Departments of Pathology, Biochemistry & Molecular Biology, College of Medicine, Pennsylvania State University, Hershey, PA 17033, USA; 2 Gittlen Cancer Research Foundation, Hershey Medical Center, Pennsylvania State University, 500 University Drive, Hershey, PA 17033, USA

**Keywords:** Aptamer, SELEX.

## Abstract

Oligonucleotide aptamers are highly structured DNA or RNA molecules, or modified versions thereof, that can bind to targets with specific affinities comparable to antibodies. They are identified through an *in vitro* selection process termed SELEX (Systematic Evolution of Ligands by EXponential enrichment) to recognize a wide variety of targets, from small molecules to proteins, and from cultured cells to whole organisms. Aptamers possess a number of desirable properties, such as ease of synthesis, stability, robustness, and lack of immunogenicity. Standard SELEX libraries require two primers, one on each side of a central random domain, to amplify the target-bound sequences via PCR or RT-PCR. However, these primer sequences cause non-specific binding by their nature, and have been reported to lead to large numbers of false-positive binding sequences, or to interfere with binding of sequences within the random regions. This review is focused on methods which have been developed to eliminate fixed primer interference during the SELEX process.

## 1. Introduction

Aptamers are oligonucleotide or peptide molecules that have the ability to bind to specific target molecules. Aptamers are usually created by selecting them from a huge random sequence pool, although some “natural” aptamers also exist (e.g. riboswitches). These molecules can be used for both basic research and clinical purposes as drugs and diagnostic sensors. Oligonucleotide aptamers can be identified through repeated rounds of *in vitro* selection (SELEX, Systematic Evolution of Ligands by Exponential Enrichment), for binding to various molecular targets such as small molecules, proteins, nucleic acids, and even cells, tissues and organisms. Peptide aptamers are combinatorial protein reagents that are usually selected from randomized expression libraries in yeast, by virtue of their ability to bind to a given target protein under intracellular conditions. Most commonly, the term aptamer refers to oligonucleotide (RNA or DNA) aptamers, which is how we use the term here. Aptamers offer molecular recognition properties that rival those of antibodies. In addition to their high specificity and binding affinity [1], they also offer many advantages over antibodies; they can be identified completely in vitro, are readily produced by chemical synthesis, possess desirable storage properties, are more robust for many processing conditions and thereby offer easy multiplexing capabilities, are much smaller than antibodies, and they elicit little or no immunogenicity in therapeutic applications.

In 1990, two labs independently developed the technique of selection [2,3]. The Gold lab coined the term SELEX for their process of selecting RNA ligands against T4 DNA polymerase. The Szostak lab, selecting RNA ligands against various organic dyes, coined the term aptamer (from the Latin word ‘aptus’ meaning to fit and Greek word ‘meros’ meaning particle) and described the process as *in vitro* selection. Two years later, the Szostak lab and Gilead Sciences, independent of one another, used *in vitro* selection schemes to evolve single stranded DNA ligands for organic dyes and the human coagulant thrombin, respectively [4,5]. There does not appear to be any systematic differences between RNA and DNA aptamers, although DNA aptamers have greater intrinsic chemical stability. In 2001, the SELEX process was automated by the Ellington lab and SomaLogic Inc, reducing the duration of a selection experiment from a few months to a few days [6,7]. Lately, the concept of smart aptamers has been introduced, which describes aptamers that are selected with pre-defined equilibrium (K_d_) and rate (k_off_, k_on_) constants and thermodynamic (ΔH, ΔS) parameters of aptamer-target interaction [8]. Kinetic capillary electrophoresis is the technology used for the selection of smart aptamers, and it yields aptamers after a few rounds of selection [9].

While the process of artificially engineering nucleic acid ligands is very useful in biology and biotechnology, the notion of aptamers in the natural world was note appreciated until 2002, when the Breaker lab discovered a nucleic acid-based genetic regulatory element called a “riboswitch” that possesses similar molecular recognition properties to artificially made aptamers [10,11]. In addition to the discovery of a new mode of genetic regulation, this added further credence to the notion of an ‘RNA World’, a postulated stage of early life on earth [12]. Recent developments in aptamer-based therapeutics have been rewarded in the form of the first aptamer-based drug approved by the US FDA for treatment of age-related macular degeneration (AMD), called Macugen offered by OSI Pharmaceuticals (http://www.osip.com). Some other companies are very active within this field, having aptamer based therapeutics and/or diagnostics under product development or in clinical trials. Examples include Archemix Inc., USA (http://www.archemix.com), Noxxon Pharma AG, Germany (http://www.noxxon.net), Isis Innovation Ltd., UK (http://www.isis-innovation.com), and SomaLogic Inc., USA (http://www.somalogic.com). In 2008, a technology for biomarker discovery termed AptaBiD (Aptamer-Facilitated Biomarker Discovery) was described for definition of molecular targets on cells, which facilitates exponential detection of biomarkers [13]. It involves three major stages: (i) differential multi-round selection of aptamers recognizing biomarkers on target cells; (ii) aptamer-based isolation of biomarkers from target cells; and (iii) mass spectrometric identification of biomarkers. The important feature of the AptaBiD technology is that it produces synthetic affinity probes (aptamers) simultaneously with biomarker discovery. In AptaBiD, aptamers are developed for cell surface biomarkers in their native state and conformation. In addition to facilitating biomarker identification, such aptamers can be directly used for cell isolation, cell visualization, and tracking cells in vivo. They can also be used to modulate activities of cell receptors and facilitate delivery of different reagents (e.g. siRNA and drugs) into the cells.

Within less than two decades, a large number of aptamer-related articles have been published (searching PubMed with the term “aptamers” yields > 1700 hits, and “DNA aptamers yields > 1200; see www.ncbi.nlm.nih.gov), and many aspects of aptamer research and development have been thoroughly reviewed, including (i) SELEX technology, which includes the general principle, starting random DNA oligonucleotide libraries, targets, selection conditions, amplification, cloning and characterization; (ii) chemical modifications; (iii) fields of application; and (iv) advantages and limitations. This review is intended to get around a central limitation of the selection process, namely ways to reduce the influence of the constant primer regions of the nucleotide libraries that they are used for identification of aptamers. While elegant computational methods have suggested that fixed primer sequences do not significantly influence the secondary structure of already-identified aptamers [14], the presence of fixed primer sequences during selection can result in many problems [15,16]. 

## 2. Primer-eliminating methods for genomic SELEX

In SELEX as originally developed [2], the library utilized contained 10^14^-10^15^ random sequences. PCR amplification requires that the nucleic acid sequences of interest to be flanked by fixed sequence primer annealing sites. A T7 promoter is included in one of the primer annealing sides so that the library can be expressed as RNA, or a biotin is linked to a primer to allow use of a single stranded DNA library. Genomic SELEX is an extension of SELEX, and provides a useful tool for identifying genomic DNA or RNA transcripts that bind tightly to target proteins *in vitro*, with the expectation that identified protein-nucleic acid interactions will provide insight into *in vivo* biological events. To yield significant results, the binding between target proteins and the genomic library should not be influenced by artificial factors, such as the presence of primer-annealing sequences of the library. One important drawback is that the diversity of the genomic libraries is limited; for example, a fully representative genomic library of *Escherichia coli* contains only ~4.6 ´ 10^6^ different genomic sequences, although it is well-suited for the identification of specific nucleic acid binding protein recognition sites.

### 2.1. Primer-annealing and primer-switching genomic RNA library selection

**Figure 1:**
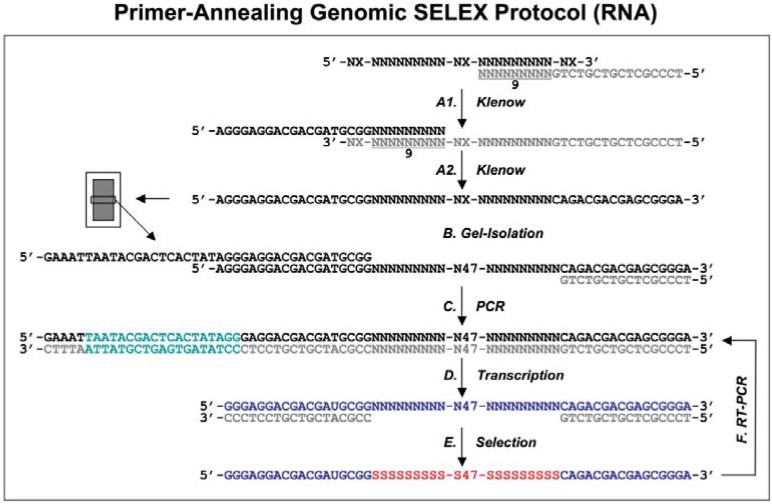
Diagram of genomic library construction and primer-annealing genomic SELEX. The 3’ primer (indicated in gray) is annealed to the denatured genomic DNA (black), and the newly synthesized strand by Klenow enzyme (A1) serves as template for second strand synthesis (A2) primed by the 5’ primer (black). The products of the reactions are run on a denaturing gel and a fraction containing ~65 nt genomic inserts is isolated (B) and then PCR amplified to generate the *in vitro* transcription templates (C; the green portion indicates a T7 RNA polymerase promoter). RNA library (blue) is transcribed (D), and subjected to the regular selection (without the complementary oligos, indicated in gray) and the primer-annealing selection (with the gray oligos) in presence of target protein (E). The target bound sequences (red) are amplified by RT-PCR for next round selection (F). This protocol is distilled from Shtatland *et al.* [16]

Shtatland *et al.* [16] first reported 2 methods (termed “primer-annealing” and “primer-switching”) using an *E. coli* genomic library constructed previously [17]. *E.coli* genomic DNA was denatured and annealed to an oligo with 9 random nucleotides (termed ‘tail’) at the 3′ end and a fixed sequence at the 5′ end, then the oligo was extended with Klenow fragment of *E.coli* DNA polymerase (Figure 1A_1_). Another randomized oligo with a different 5′ fixed sequence was added to the first reaction products, then annealed and extended in the same way (Figure 1A2).

**Figure 2:**
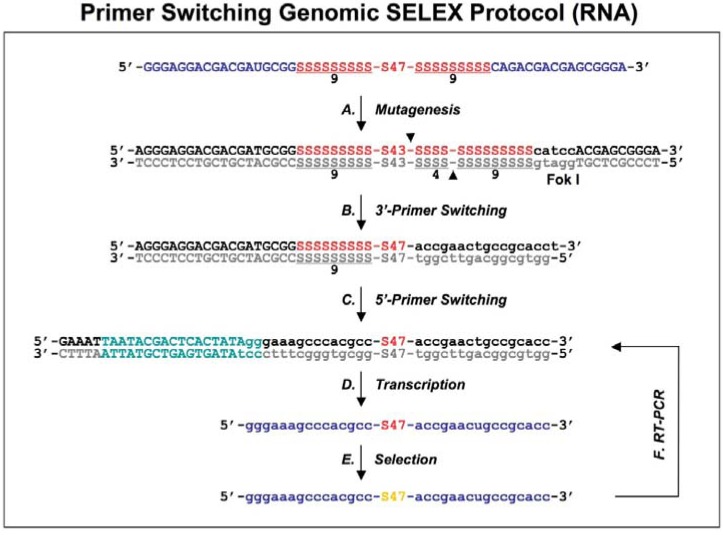
Diagram of primer-switching genomic SELEX. The third round RNAs selected by regular selection are reverse transcribed to cDNAs, and PCR amplified by 5’primer and modified 3’ primer to a introduce Fok I restriction site (indicated in lower case, with its cutting sites indicated by black arrowheads; A). After Fok I digestion the overhang at the cut end of the library DNA is extended with Klenow enzyme, and blunt-end ligated to the new 3’ fixed sequence indicated in lower case (B). The steps of A and B are repeated but this time for the 5’ end, to generate a new transcription template (C; the T7 RNA polymerase promoter is indicated in green) for the following transcriptions, selections and re-amplifications (D-F). This protocol is distilled from Shtatland *et al .* [16]

The extended reaction products were run on a denaturing gel to fractionate by size, and each fraction became the basis of a library with a different length of genomic insert (Figure 1B). The library was completed by PCR amplification that added a T7 transcription promoter to one of the primer annealing sites (Figure 1C). Finally, a library containing ~65 nt genomic inserts (9 nt tail + 47 nt genomic insert + 9 nt tail) flanked by fixed sequences, which served as primer annealing sites for amplification, was chosen for further primer-annealing SELEX (Figure 1D-F) and primer-switching SELEX (Figure 2). In each round of selection, the transcribed libraries were allowed to bind target (MS2 coat protein) and then the bound RNAs were amplified by RT-RCR.

First, a standard SELEX protocol was performed (which was same as the primer-annealing SELEX shown in Steps D-F in Figure 1, except that the complementary oligos of the fixed regions were absent during the selection process) with five rounds of selection. The second, primer-annealing SELEX, was designed to reduce the participation in binding of only the fixed sequences, but not the tails. The standard SELEX protocol was carried out for three rounds. In the three subsequent rounds, two DNA oligonucleotides complementary to the two fixed sequences were annealed to RNA prior to its binding to target protein. Switching from “no annealing” to “annealing”, rather than doing all six rounds “annealing”, should reduce the fraction of isolates that require annealing for binding to target protein. The third method, primer-switching SELEX (Figure 2), eliminated the binding artifacts by completely switching the tails and fixed sequences halfway through the course of SELEX (replacing the fixed sequences with entirely different fixed ones). A FokI endonuclease restriction site (FokI cuts at a specific distance, 9-13 nt, from its restriction site) was introduced by RT-PCR (Figure 2A) and used for cutting the 3′ fixed sequence and the tail of the library DNA selected after three rounds of regular SELEX. After digestion with FokI, the overhang at the cut end of the library DNA was extended with the Klenow fragment of *E.coli* DNA polymerase, and blunt-end ligated to the new 3′ fixed sequence, which was a duplex of synthetic oligonucleotides (Figure 2B). The ligation product was amplified by PCR and the whole procedure was repeated, this time to switch the 5′ fixed sequence (Figure 2C).

The three pools of selected DNAs obtained from the three different protocols were cloned and sequenced after five or six rounds of selection, at which point optimal binding was observed. The results showed: (i) for the regular SELEX, 90% of the GeneBank related isolates were experimentally-induced artifacts; in most of them the fixed sequence participated in forming the binding site, which also had several mutations in the tails; (ii) for the primer-annealing SELEX, 60% of GenBank related isolates were artifacts, and the fraction of artifacts in which the fixed sequences participated in binding decreased ~2-fold; and (iii) for the primer-switching SELEX, only 1 out of 77 GeneBank related isolates was artifactual. These quantitative data clearly demonstrate the potential problems which can be caused by fixed-primer sequences. 

### 2.2. Primer-free genomic DNA library selection

To completely eliminate artifacts introduced by the fixed regions (including the tails and the primers), Wen and Gray designed a primer-free genomic SELEX method [18], in which the primer-annealing sites were removed prior to the selection step and then regenerated prior to the amplification. 

**Figure 3:**
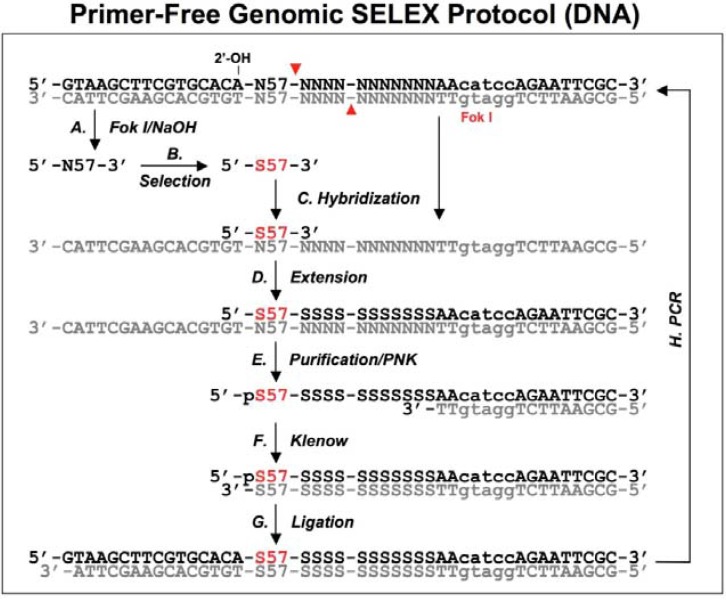
A schematic procedure for primer-free genomic SELEX. The genomic library is shown as dsDNA, at the conjunction between 5’ primer and genomic insert a ribose is indicated as 2’-OH, at the 3’ end the Fok I recognition site is indicated as lower case and its cutting sites are indicated by the red arrowheads. The starting library is generated by Fok I digestion followed by alkaline hydrolysis (A), after selection (B), it is annealed to dsDNA library (C) to synthesize the 3’ tail and fixed sequences (D). The extended products are purified and phosphorylated by T4 polynucleotide kinase, PNK (E), then annealed to 3’ primer and the overhand is filled in using Klenow enzyme (F). After ligation with the 5’ duplex the selected sequences are PCR amplified for next round of selection. This protocol follows that described by Wen and Gray [18].

A bacteriophage fd genomic library was generated by introducing a restriction site in the 3' end and a ribose linkage in the 5' end primer-annealing regions, which were removed by enzymatic and alkaline treatments respectively (Figure 3A). The tail regions, which usually contained a few point mutations due to the library construction, were also concomitantly removed by this procedure. The starting material, finally trimmed to ~57 nt and containing only genomic sequences, was used for the selection reaction with the target protein, g5p (Figure 3B). To regenerate primer-annealing sites, the selected sequences were hybridized to the unselected dsDNA templates (Figure 3C), and the hybridized inserts were then elongated by a thermo-stable DNA polymerase to synthesize the 3' primer-annealing sequence (Figure 3D). The elongated products were separated from the templates on a denaturing gel (Figure 3E), and the purified products were phosphorylated at the 5’ end by T4 polynucleotide kinase and then converted to blunt end duplexes by Klenow enzyme (Figure 3F) before ligation of a 5’ end primer duplex (Figure 3G). Finally, the selected sequences were amplified by PCR for the next round of selection (Figure 3H). After four rounds of selection, a major group of sequences was identified and the major sequence, 57 nt, was tested in comparison with a downstream sequence as a control. It showed a ~23-fold higher binding, which demonstrated that the selected genomic sequences had higher affinity for g5p binding than an unrelated neighboring sequence.

This protocol relies on minimal diversity of the library for successful hybridization. Libraries or selection pools that are more complex than the fd genomic library have to be tested for efficiency of hybridization at this step. The fd genomic DNA is only 6408 nt in length, and it is theoretically represented by about the same number of unique sequences in the library. If a more complex starting library is used, a few initial rounds of regular or other types of SELEX could be performed to reduce the diversity of the selection pool, similar to the approach used in the primer-switching SELEX [16], which would help decrease the chance of preferential selection of sequences with repeated elements, before the primer-free protocol is applied.

## 3. Primer-free 2’O-Methyl random RNA fishing SELEX

**Figure 4:**
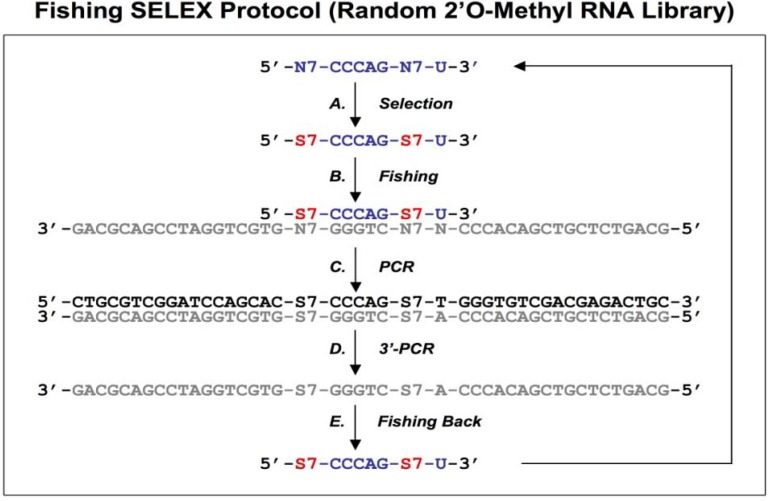
Diagram of fishing SELEX using a random 2’O-Methyl RNA library. A chemically synthesized random 2’O-Methyl RNA library (indicated in blue) is incubated with target (A), the target bound sequences allow the fishing of the complementary DNA oligonucleotides (B). The fished DNA templates are amplified by regular PCR (C), and then the bottom strand sequences (indicated in gray) are synthesized by PCR using 3’ primers only (D). The amplified bottom strand sequences allow the fishing-back of the 2’O-Methyl RNA sequences, which are used for next round of selection. This protocol is from Boiziau and Toulme [19].

Most *in vitro* selections are performed with unmodified RNA or DNA sequences, leading to aptamers of high affinity and specificity but with relatively short lifetimes *in vivo*. Only a limited number of modified triphosphate nucleotides conferring nuclease resistance to the oligomer can be incorporated by polymerases. This encourages the development of alternative methods for the identification of nuclease-resistant aptamers. Boiziau and Toulme described such a “fishing” SELEX method [19]. After selection with a random 2’O-methyl RNA library against the TAR-RNA of HIV-1 (Figure 4A), the complementary DNA sequences were fished out of a randomized DNA library by Watson-Crick hybridization (Figure 4B). The DNA-fished sequences were PCR amplified as dsDNAs (Figure 4C) and ssDNAs (Figure 4D). The ssDNAs were used to fish back the chemically modified candidates from the initial library (Figure 4E). This procedure allowed an indirect amplification of the selected candidates, and the enriched pool of 2’O-Methyl RNA was then used for the next round of selection.

Fifteen selected sequences were analyzed after six rounds of selection. No clear consensus emerged at this point of the selection although a non-statistical repartition of nucleotides was observed. Thus, this selection was still not sufficient, and no follow-up data were reported, suggesting limitations to the approach. Since a random library containing n nucleotides generates a 4^n^ diversity, this method is limited to very short random regions. In this case, a 14 nt randomized library led to a theoretical population of 2.7 × 10^8^ different molecules, which is over a million times smaller than standard starting pools of 10^14^-10^15^ molecules. 

As a potential way to bypass these limitations, one option would be to perform a few rounds of selection with unmodified RNA pools; once some of convergent positions are identified, the chemically modified library could be synthesized to contain the convergent (fixed) nucleotide positions separated by a few short random regions. This restricted 2’O-Methyl RNA library might prove useful for further, more efficient selections.

## 4. Primer-eliminating methods for random RNA SELEX

### 4.1. Primer-less random RNA library selection

While the described genomic SELEX is only useful for identification of certain genomic sequences, and the fishing SELEX appears to have only limited applicability, the Klussmann lab [20] developed an integrated method to identify aptamers with 10 fixed nucleotides through ligation and removal of primer binding sites within the SELEX process. This primer-less SELEX, also termed tailored-SELEX, results in short sequences that can be tested more rapidly in biological systems. The identified aptamer was validated by identifying a Spiegelmer (“mirror-image aptamer”) that inhibited the action of the migraine-associated target α-CGRP (calcitonin gene-related peptide 1) with an inhibition constant (IC_50_) of 3 nM in cell culture [20]. This Spiegelmer also reduced electrically-evoked increases in meningeal blood flow in a dose-dependent manner, but unexpectedly also reduced the CGRP release caused by electrical stimulation in a hemisected skull preparation [21].

**Figure 5:**
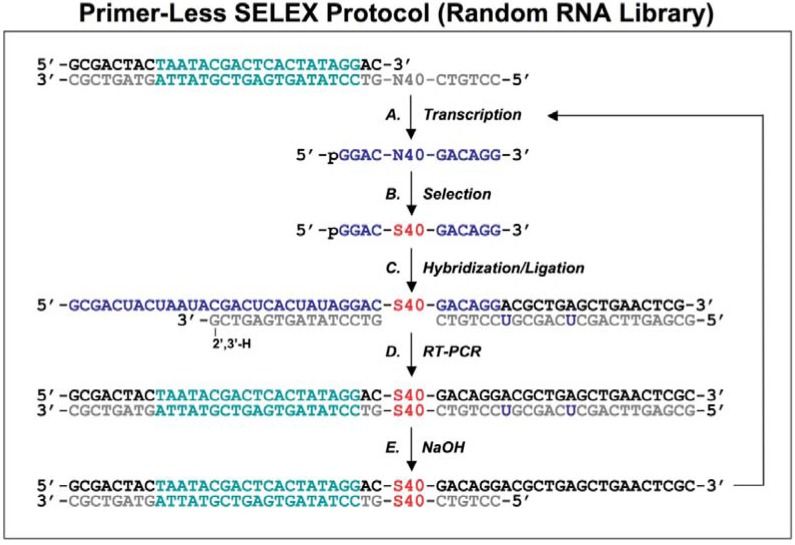
Diagram of primer-less random RNA SELEX. The *in vitro* transcription templates are generated by annealing 2 oligonucleotides together, in which a portion of the templates is T7 RNA polymerase promoter (shown in green). The starting RNA library (blue) is transcribed (A) and selected by incubation with target protein (B), and then the selected RNA sequences are ligated with 5’ and 3’ duplexes (C) and RT-PCR amplified with 5’ primer containing a T7 promoter and 3’ primer containing a 2’ ribose (indicated in blue; D). The amplified products are subjected to alkaline hydrolysis to generate the transcription templates for the next round of selection. This protocol depicts that described by Vater *et al.* [20]

In this method, a 50 nt core library contained a 40 nt randomized region plus a total of 10 fixed flanking nucleotides. It was transcribed from the template that was constructed by annealing two oligonucleotides together; the fixed regions are necessary for an efficient ligation of the primer binding sites that are needed for amplification (Figure 5A). After selection with D-α-CGRP (Figure 5B), two oligonucleotides (referred to as ligates) were enzymatically ligated to the selected core library with the assistance of bridging oligonucleotides (bridges) that are complementary to one of the ligates and the respective fixed sequence of the RNA library (Figure 5C; see the original article for details). After RT-PCR amplification (Figure 5D), the PCR products were cleaved under alkaline conditions, leading to unequal length strands. The bottom strand lacked the unwanted 3’ primer region, and served as a template for further *in vitro* transcription (Figure 5E).

The enriched pool obtained after 15 rounds of selection was cloned and 38 sequences were determined. Four motifs were identified by sequence alignment, which occurred in every sequence, either alone or in combination with each other. 9 sequences were synthesized as Spiegelmers, which blocked rat α-CGRP-induced cAMP formation with an IC_50_ of 500 nM or better in cell culture. The best one showed an inhibition with an IC_50_ of 3 nM, and as a control the inverse sequence showed no inhibitory effect. While the 5’ fixed sequence was needed for target binding, the six 3’ terminal fixed nucleotides could be removed, and the resulting truncated Spiegelmer had the same inhibition potency as the full-length Spiegelmer.

### 4.2. Dual random RNA library selection

The above primer-less SELEX is based on a ligation strategy for the primer binding site to an RNA library carrying a small number of fixed nucleotides on both sides of the randomized region. Compared to a standard SELEX only two additional reaction steps are needed: alkaline hydrolysis and ligation, the latter of which might result in missing some of the selected sequences due to low yields of ligated products, which could be problematic, especially for the initial round(s) of selection. Nevertheless, an automation protocol for the so-called “tailored” SELEX-process is possible [22] although it is significantly slower than automation processes that employ conventional protocols [7,22]. 

**Figure 6:**
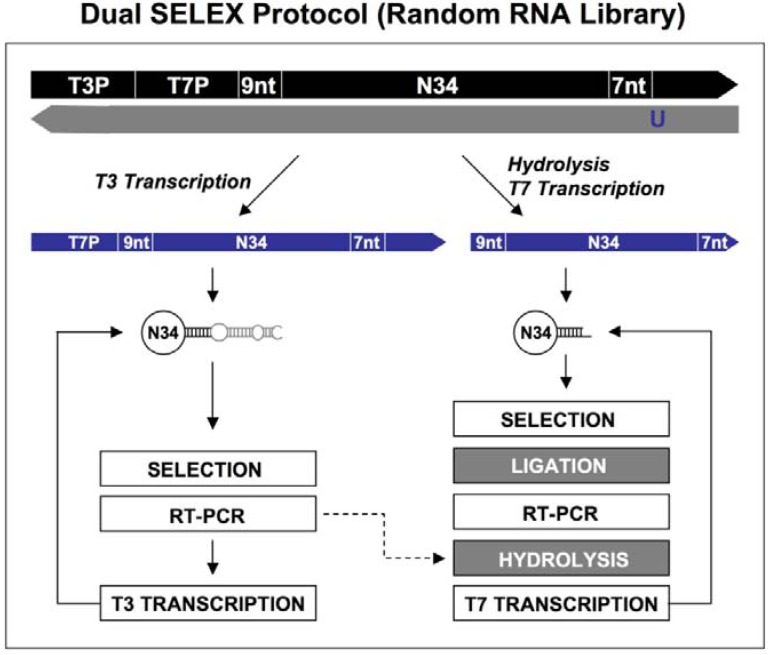
Diagram of dual SELEX. Tandem promoter sequences for T3 and T7 RNA polymerases are located at the 5' end of the library. The integration of the reverse primer that contains ribose (blue U), introduces the cleavage sites for alkaline hydrolysis of the template. The full-length RNA library is transcribed by T3 RNA polymerase, and the short RNA library is generated by using T7 RNA polymerase and the alkaline-hydrolyzed templates. The templates of the PCR products are shared templates that can be switched, commonly after a few rounds of full-length RNA library selection, which can be switched to short RNA library selection as the dashed arrow indicated. This protocol is from Jarosch *et al.* [23]

To overcome the limitations mentioned above, Jarosch *et al.* modified their primer-less library to a dual RNA library (Figure 6) that can be used in two different formulations: as a (conventional) full-length library with primer binding sites or as a short library without primer binding sites [23]. The library carries seven complementary (fixed) nucleotides that clamp the randomized region and constrain the oligonucleotides into a partly double-stranded structure. This design already minimizes the risk that the primer binding sites become part of the target-binding motif. Moreover, the sequence arrangement of the dual library also allows one to carry out the selection with the short library so that the flanking primer binding sites are partially removed before the selection step and added back subsequently. It seems likely that the secondary structure of the constrained nucleotides might introduce some additional complications.

In order to limit potential losses of valuable target binding sequences due to additional enzymatic steps, in early selection rounds only the full-length library was employed by using a faster (automated) selection protocol. After a pool of target-binding (full-length) sequences was achieved (nine selection rounds), the selection protocol was switched to the short library for another nine rounds of selection. All selected sequences shared a common sequence that was already described as a ghrelin-binding motif “NOX-B11”, and a newly selected Spiegelmer (NOX-B11-3) inhibited activation of the GHS-R1a receptor displaying an IC_50_ of 4.5 nM, which is ~5-fold lower than the IC_50_ of Spiegelmer NOX-B11 previously selected from the full-length library [24]. In electrophysiological *in vitro* single cell recordings, NOX-B11-3 effectively blocked the excitatory effect of ghrelin in the medial Arc (ArcM) of rats and potently suppressed ghrelin-induced c-Fos expression in the ArcM of rats [25]. However, the same problem was observed as with the primer-less SELEX; one side of the short primer regions could not be removed, and thus contributed to the binding site. However, the dual SELEX speeded up the process by allowing partial employment of the automated selection protocol. 

## 5. Methods for reducing/eliminating primers for random DNA SELEX

We developed two DNA-based methods that reduce and/or eliminate the primer sequences from the target-binding step, thus reducing or eliminating the interference with the selection process caused by the primer sequences [26,27]. In these methods, the starting selection libraries contain a central random sequence that is: (i) flanked by only 2nt on each side (minimal-primer); or (ii) flanked by no fixed sequences (primer-free). These methods allow primer regeneration and re-elimination after and before selection and don’t require any chemical modifications for selection in a variety of conditions. Further, the selection rounds are performed with DNA oligomers, which can be employed as end product aptamers. 

### 5.1. Minimal-primer random DNA library selection

**Figure 7:**
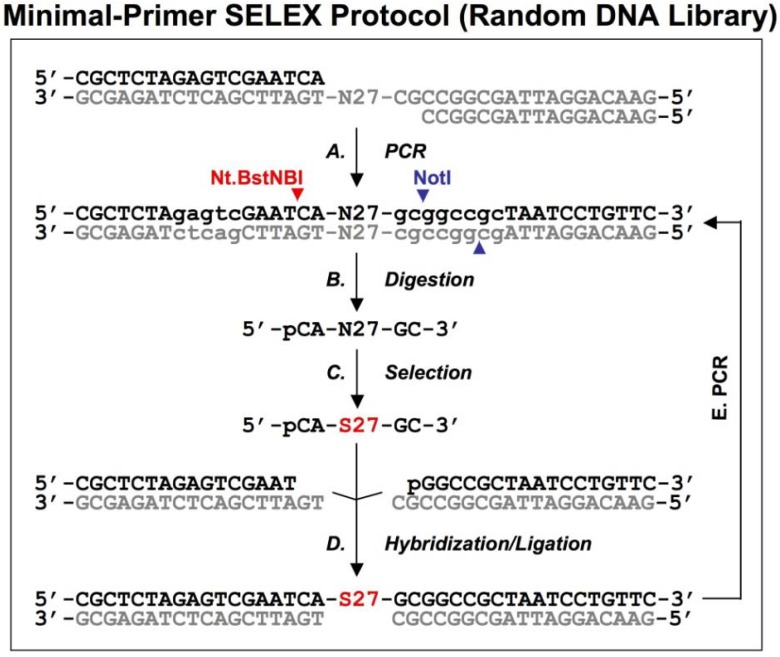
Schematic representation of the minimal-primer random DNA library selection. Oligonucleotides are annealed together, and subjected to PCR amplification to yield a dsDNA library (A). The primer-less ssDNA library is from Nt.BstNBI /NotI digestions and gel-purification (B), the Nt.BstNBI and NotI recognition sequences are indicated in lower case and their cutting sites are indicated by red and blue arrowheads, respectively. After selection (C), the selected library is ligated with 5’ and 3’ duplexes to re-generate the primer regions (D), and finally re-amplified by PCR (E) for the next round of selection.

The dsDNA library was constructed using PCR with the corresponding oligonucleotides (Figure 7A), which contain a central random domain of 27 nt flanking two primer regions. In the 5’ region, it contains a ‘nicking’ site for endonuclease Nt.BstNBI; this enzyme recognizes dsDNA but cleaves only one strand of the DNA substrate. In the 3’ region, it contains a NotI endonuclease restriction site that cleaves dsDNA substrate. A starting library, 31 nt in length, is generated by NotI/Nt.BstNBI cleavage of the dsDNA followed by gel-purification. As shown, this produces the random N27 sequences with 2 nt flanking sequences (“docks”) on each side (Figure 7B). The 31 nt-fragments with the 2 nt docks are gel-purified after the digestion and used for selection. Following the selection (Figure 7C), the bound 31 nt-fragments are used for primer regeneration (Figure 7D) to allow reamplification of the selected sequences for the next round of selection (Figure 7E).

### 5.2. Primer-free random DNA library selection

**Figure 8:**
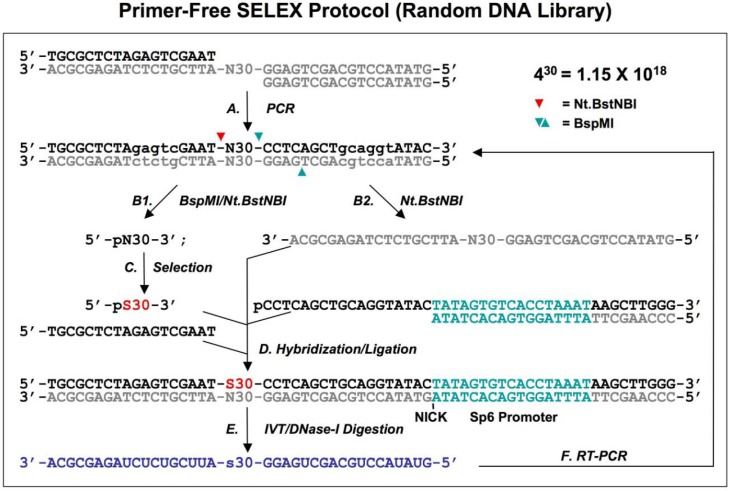
Schematic representation of the primer-free random DNA library selection. Oligonucleotides are annealed together, and subjected to PCR amplification to yield a dsDNA library (A). The primer-free ssDNA library is isolated by Nt.BstNBI/BspMI digestions and gel-purification, and the self-bridge (indicated in gray) is from single-digestion of Nt.BstNBI (B), the Nt.BstNBI and NotI ercognition sequences are indicated in lower case and their cutting sites are indicated by red and green arrowheads respectively. After selection (C), the selected sequences are hybridized with their corresponding oligomers and the self-bridge, and ligated to re-generate the primer regions and an extra Sp6 promoter (indicated in green) at the 3’ end (D), and then transcribed to RNA (indicated in blue; E) and finally re-amplified by RT-PCR (F) for next round of selection.

The library construction for this method is the same as for the minimal-primer library (Figure 8A), but it contains a central random domain of 30 nt. This is flanked by the 5’ region containing a “nicking” site for the endonuclease Nt.BstNBI, and the 3’ region containing a BspMI endonuclease restriction site. The BspMI enzyme cleaves both strands, leaving 4 additional 3’ nucleotides. The 30 nt of 5’-pN30-3’ fragments, and the full-length lower strands which serve as “self-bridge” fragments, are generated respectively by BspMI/Nt.BstNBI and Nt.BstNBI cleavage of the dsDNA library followed by gel-purification (Figure 8B). After selection (Figure 8C), the unbound fragments are washed away, and the bound selected fragments are used for re-generation of the primer regions. 

For the hybridization/ligation reaction, the self-bridge was produced and purified at the same time as the 30 nt fragment purification. The 5’ primer is the same as for the library construction, and the 3’ primer is replaced with matching primer that also contains an additional Sp6 transcription promoter at the 3’ end (Figure 8D). The products of the hybridization/ligation reaction are then used for *in vitro* RNA transcription (Figure 8E). Following the RNA transcription, the ligated DNAs (including the unselected self-bridge DNA fragment) are digested by DNase I to remove interfering background sequences. The reverse transcription (RT)-PCR data have shown that the primer-regenerated products were efficiently reamplified for next round of selection (Figure 8F).

**Figure 9:**
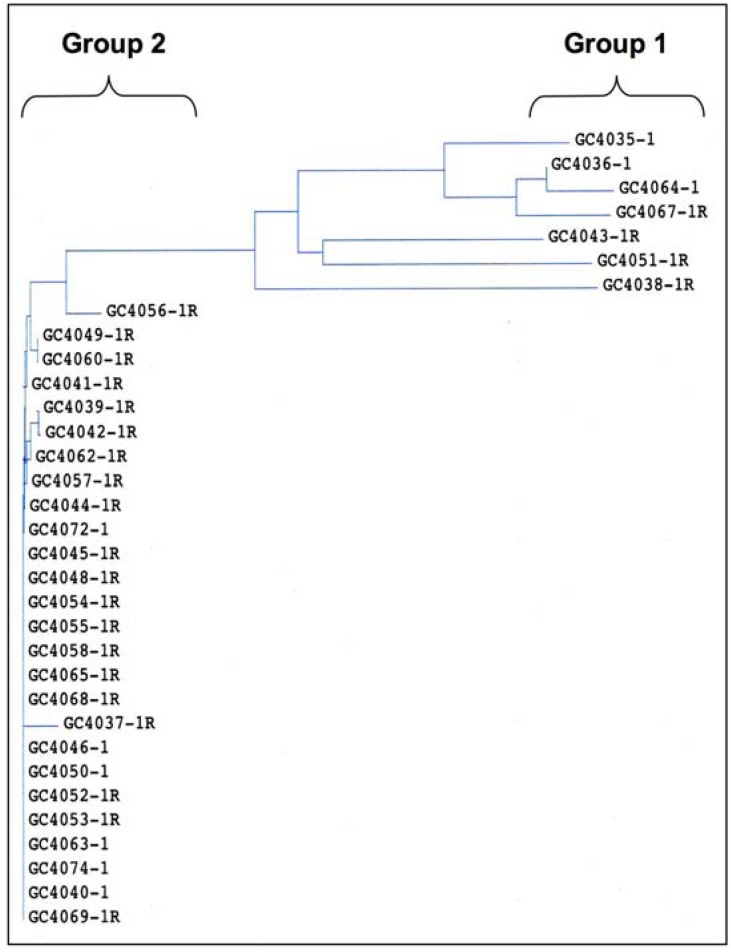
Schematic representation of aptamers selected using the primer-free random DNA library protocol. Selection was performed for 10 rounds, and selected aptamers were cloned and sequenced. Analysis of the sequences showed that they fell into 2 distinct groups. Binding experiments with radioactivity-labeled consensus sequences showed that binding of the 2 consensus sequence groups was additive, indicating that they targeted distinct sites. Binding parameters are currently being characterized and will be reported elsewhere.

We have used both the minimal-primer and primer-free protocols to identify DNA aptamers which bind to human S100b protein (Gene ID# 6285), a clinically relevant serum marker for melanoma. After 10 rounds of selection, ~30-50 selected aptamers were cloned and sequenced. A limited number of conserved sequence families were identified by each technique. For example, the primer-free method (from Figure 8) identified 2 basic consensus sequence families (Figure 9). 

The Group 2 family contained 25 sequences with extremely high homologies over 26 nt, including a completely conserved central block of 11 nt. The Group 1 family was also closely interrelated and targeted a distinct site on S100b (since binding of labeled aptamers was additive). With the minimal-primer protocol, 5 basic sequence families were identified from 38 isolates, which also contained closely interrelated members. Details regarding characterization of the selected aptamers will be reported elsewhere, although they show very good binding properties.

## 6. Conclusions

This review discusses a series of modifications to standard SELEX protocols which have been devised to eliminate problems caused by fixed-primer sequences flanking random libraries. Methods for use of genomic libraries were devised for various purposes (for example, identification of binding sites for DNA binding proteins). While well-suited to the tasks they were developed for, a general limitation is the limited diversity of the genomic libraries. Other techniques (e.g. Figure 4) have been developed to allow identification of RNA aptamers with various substitutions designed to enhance their in vivo stability. Further techniques (e.g. Figure 5 and Figure 6) were developed for RNA aptamer selection with considerably reduced fixed-primer sequences. Curiously, both techniques described actually identified aptamers whose binding properties were dependent upon one side of the shortened fixed-primer regions. Identified RNA aptamers still require chemical modifications to enhance their stability, so that modified aptamers either need to be “fished out” of random libraries containing modified nucleotides, or alternatively modified aptamers need to be re-tested for binding properties. Finally, minimal-primer and primer-free protocols have been published which allow selection of aptamers from random DNA libraries. These should directly provide inherently more stable aptamers, and relatively simple modifications can be introduced for enhanced stability. To briefly summarize , primer-eliminating methods for SELEX have afforded clear advantages: (i) Primer-eliminating methods for genomic SELEX have been used to precisely identify protein binding sequences in a genomic DNA library, although the libraries of necessity have limited diversity; (ii) Primer-free 2’O-Methyl random RNA fishing SELEX was a unique method, which used a fully chemically-modified library to carry out SELEX process, which directly resulted in nuclease-resistant aptamers; however, the diversity of the library was quite limited, and only some modifiable nucleotide positions were identified, with no consensus sequences forthcoming; (iii) Primer-eliminating methods for random RNA SELEX have been successfully used to identify two aptamers and their mirror imagers “spiegelmers” were significantly active in cells; the libraries were flanked by at least ten fixed nucleotides that could not be fully eliminated; (iv) Primer-eliminating methods for random DNA SELEX have completely eliminated the primer regions from random libraries during the selection process, and have been used to generate consensus aptamers in the absence of fixed sequences. 
